# Malignant Mixed Germ Cell Tumors of the Ovary: A Series of Rare Cases

**Published:** 2019

**Authors:** Lajya Devi Goyal, Balpreet Kaur, Rama Kumari Badyal

**Affiliations:** 1- Department of Obstetrics and Gynaecology, Guru Gobind Singh Medical College and Hospital, Faridkot, Punjab, India; 2- Department of Pathology, Guru Gobind Singh Medical College and Hospital, Faridkot, Punjab, India

**Keywords:** Alfa feto protein, Endodermal sinus tumor, Malignant mixed germ cell tumor

## Abstract

**Background:**

Malignant mixed germ cell tumors of ovary are rare aggressive cancers affecting young adolescent girls. The commonest combination reported in literature is dysgerminoma and endodermal sinus tumors but in our study the most common combination was immature teratoma and endodermal sinus tumor which is exteremely rare. Preservation of future fertility is a concern. Fertility sparing surgery followed by combination chemotherapy is the current treatment of choice but treatment must be individualized depending upon the nature of the tumor.

**Methods:**

A retrospective study on five patients with these tumors was conducted on patients at Guru Gobind Singh Medical College and Hospital (Punjab, India) between September 2009 to January 2018.

**Results:**

Median age of patients was 15.6 years. Histopathological combination was immature teratoma and endodermal sinus tumor (n=3), endodermal sinus tumor and embryonal carcinoma (n=1), and mature and immature teratoma (n=1). Tumor markers AFP, beta HCG and LDH were raised in all except the patient with mature and immature teratoma. All patients underwent surgery followed by combination chemotherapy. Three patients developed metastasis within six months of treatment and died. In the remaining two, no reccurrence was reported till date.

**Conclusion:**

Malignant mixed germ cell tumors of ovary are extremely rare tumors and have poor prognosis. Fertility preservation is a concern as these patients are usually young adolescent girls but fertility sparing treatment must be individualized on the basis of tumor type, surgical staging, and availability of combination chemotherapy. Considering high recurrence rate and mortality, total hysterectomy with bilateral salpingo-oophorectomy with complete surgical staging followed by combination chemotherapy should be perfomed at advanced stage and aggressive tumor biology. Preservation of fertility must be held secondary.

## Introduction

Worldwide ovarian cancer accounts for 225,000 new cases and 140,000 deaths every year (1). Ovarian germ cell tumors account for 15–20% of all ovarian malignancies and incidence of malignant ovarian germ cell tumors is 2–6%. These tumors typically occur in adolescent girls and young women (2). Malignant germ cell tumors are derived from primordial germ cells of the embryonic gonad (3). World Health Organization (1973) clasified malignant germ cell tumors as dysgerminoma, endodermal sinus tumor (yolk sac tumor), and immature teratoma, non-gestational choriocarcinoma, embryonal carcinoma, and mixed germ cell type (4). Malignant mixed germ cell tumor is a type of tumor that consists of two or more malignant germ cell components. These tumors are quite rare cancers, seen in 8% cases of germ cell tumors but are very aggressive in nature (5). The most common combination reported is dysgerminoma and Endodermal Sinus Tumor (EST) (6) and the rarest combination has embryonal carcinoma and immature teratoma as its components (4). Embryonal carcinoma, although very rare, holds the most malignant potential (7). Debulking surgery is the mainstay in treatment of ovarian cancer. Since these tumors affect young adolescent girls, preservation of fertility is a major concern. Fertility preserving surgery followed by combination chemotherapy is the preferred treatment modality followed worldwide.

A study was retrospectively conducted on five patients with malignant mixed germ cell tumors of the ovary and the clinicopathological features, prognostic factors and treatment outcomes were evaluated.

## Methods

A total of five patients with malignant mixed germ cell tumors of the ovary were encountered among 528 patients of ovarian tumors from September 2009 to January 2018 in the department of Obstetrics and Gynecology, Guru Gobind Singh Medical College and Hospital, Baba Farid University of Health Sciences. The data was retrieved from central record room after the permission from Medical Superintendant and ethical committee. The age, parity, presenting features, duration of symptoms, clinical features, imaging, tumor marker levels (CA 125, CEA, AFP, LDH and βHCG), operative findings, size and site of residual disease, complications and the histological types including grade in immature teratoma were recorded. Chemotherapy regimen and its complications were also recorded. Response to treatment was assessed by evaluating clinical improvement, size of residual disease using imaging modalities and tumor markers levels. Optimal cytoreductive surgery was the aim in advanced disease. In young women where conservation of fertility was a consideration, a full surgical staging procedure and unilateral salpingo-oophorectomy was the treatment of choice in stage I disease. In cases where fertility preservation was not a concern, total abdominal hysterectomy with bilateral salpingo-oophorectomy with complete surgical staging was performed. Postoperative chemotherapy was offered to all patients. Combination chemotherapy using Bleomycin, Etoposide, Cisplatin (BEP) was given for 3–4 cycles.

AFP and beta-hCG levels were serially measured, prior to each chemotherapy cycle, after the completion of treatment to monitor the treatment response. The levels were followed every 1–2 month for 1 year after treatment, then quarterly for two years, and six monthly thereafter. Patients were examined at each follow up visit. Pelvic ultrasound was done three times monthly and CT scan was utilized when clinically indicated or in patients with rising tumor markers.

### Ethical Consideration:

Permission for the study was taken from the medical superintendant and ethical committee. Since it was a retrospective study, informed consent was taken from the patient at the time of follow up/telephonically.

## Results

In eight years period, 528 cases of ovarian malignancy were operated in our hospital. Out of these, there were five cases of malignant mixed germ cell tumors of the ovary. As shown in table 1, most common histological subtypes were endodermal sinus tumor and immature teratoma (n=3) followed by mature and immature teratoma (n=1), endodermal sinus tumor and embryonal carcinoma (n=1) (Figure 2).

**Figure 1.**
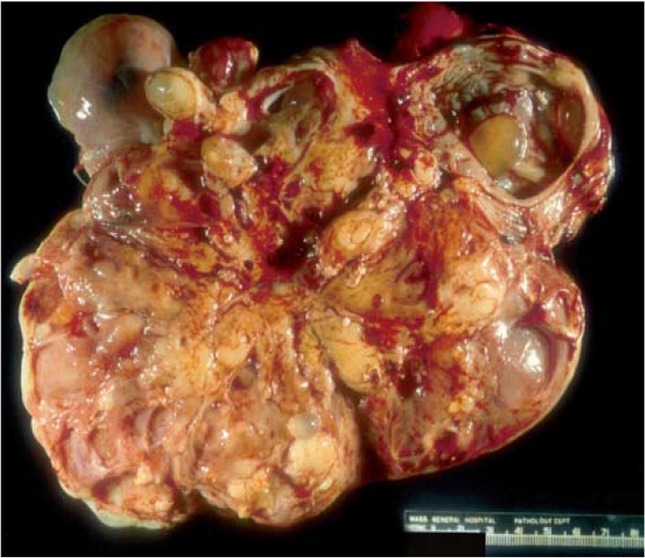
Gross photograph of malignant mixed germ tumor: The solid fleshy cut surface with few cysts along with areas of necrosis and haemorrhage

**Figure 2.**
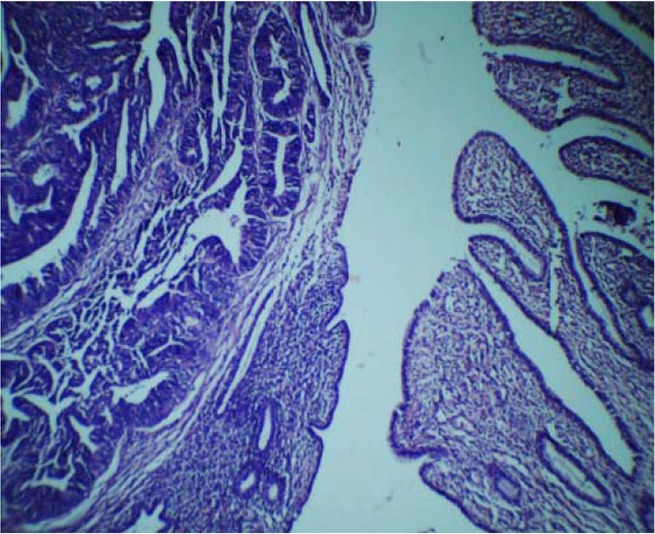
Photomicrograph showing malignant mixed germ cell tumor: Embryonal carcinoma mixed with immature neuroepithelium (left) and mature teratomatous component in the form of mucinous epithelium (right) (H & E, 100X)

**Table 1.** Clinicopathological details and follow up

**Patient identity**	**Tumor type**	**Age in years**	**Clinical features**	**Tumour markers**				**CECT findings**	**DATE of surgery**	**Surgical staging and type of surgery**	**Chemo therapy**	**Follow up**

				**AFP**	**HCG**	**LDH**	**CA125**					
**P1**	Immature+ Mature teratoma	18	Abdominal pain, ascitis	-	-	-	-	35 *cm* right ovarian mass, ascitis	13/09/2009	Stage III ULSOP, debulking	Refused	Died within 12 months of surgery
**P2**	Immatuer teratoma+EST	08	Abdominal pain, ascites, breathless- ness with pleural effusion	299	56	-	-	16 X 16 *cm* right sided abdominopelvic mass with ascitis, pleural effusion	10/09/2010	Stage IIIc ulsop with debulking residual disease >2 *cm*	BEP	Died within 6 months of surgery
**P3**	Immature teratoma+EST	14	Abdominal pain, ascitis, irregular menstruation	189	-	-	-	20 *cm* right ovarian mass with ascitis	17/03/2011	Stage Ic ULSOP	BEP	NED at 2 years of follow up
**P4**	Immature teratoma+EST	20	Abdominal pain, ascitis, breathless- ness with pleural effusion	281	-	-	-	20 *cm* right ovarian mass, ascitis, pleural effusion	13/09/12	Stage III TAH BSO, debulking	BEP	NED at 1 year 6 months of follow up
**P5**	EST + embryonal carcinoma	18	Abdominal pain, ascitis, irregular menstruation	4899	3175	-	361	30 *cm* right sided TUBO-ovarian mass with ascitis	18/10/15	Stage Ic ULSOP	BEP	Died within 6 months of surgery

The median age at presentation was–15.6 years (range 8 to 20 years). Two patients were premenarcheal. All the patients were unmarried and were not sexually active. The main presenting symptoms were abdominal pain and abdominal distension in all patients and breathlessness in two patients. Majority (75%) of the patients presented within three months of the symptoms emergence. Menstrual history revealed irregular bleeding pattern in two patients. On initial examination, all the patients had abdominopelvic mass. Ascites was present in all five patients and pleural effusion was seen in two patients. Two patients were severely anaemic with hemoglobin <6 *g/dl*.

Ultrasonography and Contrast Enhanced Computed Tomography (CECT) scan was done in all patients. Tumor was found to be unilateral and right sided in all the cases. Pleural effusion was present in two cases. Retroperitoneal lymphadenopathy was present in four cases. Tumor markers of AFP, beta hCG LDH, and CA125 were measured in all patients. Tumor markers were negative in the patient with immature teratoma and mature teratoma (P1). AFP was raised in patients with endodermal sinus tumor (189.6 *ng/ml*–940 *ng/ml*). The patient with combined endodermal sinus tumor and embryonal carcinoma had very high levels of preoperative AFP (4899 *ng/ml*), beta hCG (3751 *IU/L*) and CA125 (259 *U/ml*).

All the patients were taken up for surgery at Guru Gobind Singh Medical College and Hospital. Dates of surgery were P1- 10/09/2009, P2- 06/09/2010, P3- 17/03/2011, P4- 13/09/12, and P5- 18/10/15. Debulking with unilateral salpingo-oophorectomy with retroperitoneal lymphadenectomy was performed in four patients (P1, P2, P3, P5) and one of the patients (P4) had TAH BSO (Total abdominal hysterectomy with bilateral salpingo-oophorectomy) with debulking with pelvic lymphadenectomy as shown in table 1. P1 and P2 underwent pelvic and para-aortic lymphadenectomy, as the nodes were palpable intraoperatively. P3, 4, 5 had undergone pelvic lymphadenectomy as part of surgical staging. There were no significant intraoperative and postoperative complications. Surgical staging was stage 1c (P3, P5), stage III (P1, P2, P4). One of the patients (P2) had residual disease of >2 *cm*.

All except one patient (P1) underwent adjuvant chemotherapy with BEP. Patient with immature and mature teratoma had refused chemotherapy. She had developed multiple intra-abdominal metastasis within six months of the surgery and died. On follow up, two patients (P2, P5) reported recurrence within six months of the surgery in spite of chemotherapy. These patients died despite all second line treatment modalities. Only two patients (P3, P4) had survived with no evidence of disease on two years follow up (Table 1).

## Discussion

Supporting the rarity of the disease, the incidence of malignant mixed germ cell tumors in our study was found to be 0.9%. Unlike epithelial ovarian malignancies, malignant ovarian germ cell tumors grow rapidly and are usually symptomatic earlier. This may account for early diagnosis in the majority, at stage I, but in our study only 40% patients (n=2) presented at stage I while 60% (n=3) presented at stage III.

Dysgerminoma and EST is the most common combination reported in literature which makes one third of the total cases of mixed germ cell tumors (6) but extremely rare combination of immature teratoma and endodermal sinus were found in 60% of our patients (n=3). Only anecdotal case reports are seen in literature with this combination.

These tumors can occur at any age, but peak incidence is in adolescent age group. In the present study, average age at presentation was 15.6 years.

Presenting symptoms in such patients are painful abdomen, abdominal distention or mass in abdomen. Acute symptoms are seen in approximately 10% of patients, which may be due to tumor rupture, haemorrhage or torsion (7,9). Similar findings were shown in this study as well. Patients having embryonal carcinoma as one of the components of malignant mixed germ cell tumor might present with precocious puberty and irregular bleeding pattern, as these tumors secrete estrogens (4). Two of our patients also presented with irregular bleeding, and one of them had embryonal carcinoma. None of the patients had precocious puberty.

The tumor markers -βhCG, AFP and LDH are useful in diagnosis, prognosis, assessing therapeutic response and detecting tumor recurrence. The current Royal College of Obstetricians and Gynaecologists (RCOG) Guideline for ovarian mass in premenopausal women recommends determination of serum LDH, AFP and βhCG levels in all women under 40 years. The National Academy of Clinical Biochemistry guideline, recommends that AFP and βhCG levels should be measured whenever there is suspicion of GCT in women younger than 40 years (10). AFP or βhCG is elevated in 85% of patients with these tumors but in only 20% of patients with stage I disease. Hence, these markers have no role in screening. AFP >10,000 *ng/ml* or beta-hCG levels greater than 50,000 *mIU/ml* at initial diagnosis indicate a poor prognosis, with a 5-year survival rate of 50% (11).

In our series, all the patients with endodermal sinus tumor component, in mixed form had raised level of tumor markers. Also, the levels were significantly high in patients with embryonal carcinoma component. Tumor markers became negative in those with clinical and radiological remission (P3, P4) and were persistently high in those with recurrence (P2, P5). Regression of tumor marker is a good predictor of prognosis.

Conservation of reproductive potential is of great concern as most of the patients are in young age group. Since, these tumors are predominately unilateral and confined to one ovary in two thirds of cases, this allows for conservative, fertility sparing surgical treatment, with the addition of careful staging (12). The standard treatment protocol is unilateral adnexectomy, omentectomy, peritoneal washings, peritoneal biopsies and retroperitoneal lymphadenectomy followed by adjuvant chemotherapy. The safety of unilateral oophorectomy non-malignant germ cell tumors has been supported by many studies (12, 13). Since these tumors are highly sensitive to chemotherapy, Nishio et al. demonstrated that conservative, fertility sparing surgery is appropriate as long as chemotherapeutic agents are employed (14). Even in patients with bulky metastatic disease, a normal appearing uterus and contralateral ovary may be preserved allowing for future fertility options if desired. Patients with bulky disease in the abdomen, pelvis, and retroperitoneum should be surgically cytoreduced to optimal residual disease if at all possible (15).

In our study, all the patients had right sided ovarian involvement and on the basis of results of previous studies, fertility-conserving right-sided salpingo-oophorectomy with complete surgical staging in four patients had been performed. The other ovary was normal looking even in advanced cases. One of the patients (p4), an unmarried girl, underwent total abdominal hysterectomy with bilateral salpingo-oophorectomy as she did not want to seek future fertility. The major reason to perform a fertility sparing operation was the willingness of patients to preserve fertility. Although optimal debulking is considered as an important prognostic factor, role and extent of cytoreductive surgery remains controversial despite its routine use.

Considering the results of many studies (12, 13, 16, 17) and the chemotherapy-sensitive nature of the disease, an adequate attempt at maximal cytoreduction without compromising future fertility seems a reasonable surgical approach at present. But contrary to previous literature, in our study, two out of three patients with stage III disease and one out of two patients with stage I disease died where fertility was preserved with optimal debulking. The reason may be that one of the deceased patients had refused chemotherapy (p1) and developed recurrence. In another patient (p2), the tumor was at the advanced stage (stage IIIc) with residual disease >2 *cms*. In one patient (p5), despite being an early stage (stage Ic) disease, the tumor type (Embryonal carcinoma) was with highest malignant potential. This suggests aggressive nature of these tumors and needs for more radical surgical approach.

Surgery followed by combination chemotherapy has greatly improved the survival rates. Before the advent of combination chemotherapy, survival rate after surgery in stage I disease was about 5–20% (18). In 1980, an etoposide containing regimen, BEP (Bleomycin, Etoposide, Cisplatin) was introduced that proved to be efficacious and relatively nontoxic. A recent report from an Italian study has reported an overall survival rate of 88.8% in 123 Malignant Ovarian Germ Cell Tumors (MOGCT) patients (19). BEP regimen is now the first-line chemotherapy for malignant germ cell tumors of ovary (20).

Although randomized controlled trials on malignant germ cell tumors are lacking but several prognostic factors have been reported in retrospective studies. Lai et al. analyzed the prognostic factors of 93 MOGCTs, and suggested that tumor histology (dysgerminoma *vs*. non-dysge rminoma, p<0.0001) and the FIGO stage (I–II *vs*. III–IV, p=0.001) were significantly associated with treatment failure, whereas the histology (dysgerminoma *vs*. non-dysgerminoma, p<0.0004), the residual tumor after the salvage surgery (≥1 *cm vs*. <1 *cm*, p=0.0014), and the salvage high-dose chemotherapy failure (p= 0.0405) significantly influenced the overall survival (16).

Kumar et al. suggested lymph node involvement as an independent predictor of poor survival (17). On the other hand, Mahdi et al. compared 493 MOGCT cases who underwent lymphadenectomy with 590 cases who did not have lymphadenectomy. They found that neither lymphadenectomy nor lymph node metastasis was an independent prognostic factor for survival (21). Pectasides et al. declared that if tumor appears to be confined to ovaries or pelvis, a pelvic and para-aortic lymphadenectomy should also be performed. If upper abdominal involvement is there, an effort to remove all visible tumor should be made. Also, in view of the excellent chemosensitivity, clinical judgment can be used if surgical management would significantly increase postoperative morbidity (3).

Studies on non dysgerminomatous tumors suggest, an early stage minimal residual tumor after the initial surgery, less than 100 *ml* of ascites, platinum-based chemotherapy, an AFP level less than 1,000 *kU/L*, and a non-EST histology were found to be significantly related to a more favorable prognosis (12, 17 , 19, 22, 23).

Cicin I et al. in their study on yolk sac tumors reported age, histology (mixed *vs*. pure), stage, tumor size, ascites, and marker levels were not found as prognostic factors and the presence of residual tumor (p=0.014) and BEP chemotherapy (p=0.016) were significant prognostic factors in univariate analysis (23). Recurrence was associated with poor prognosis (19, 24). In our study, delayed reporting in advanced stage, residual disease, refusal to chemotherapy and recurrence were associated with poor prognosis.

## Conclusion

This study highlighted that advanced stage at presentation and presence of tumor type with high malignant potential results in low survival rate in spite of multimodality treatment with surgery and combination chemotherapy. Owing to the aggressive nature of these tumors and high mortality rate, concerns regarding preservation of future fertility must be kept secondary. Total abdominal hysterectomy with bilateral salpingo-oophorectomy with complete surgical staging followed by combination chemotherapy must be the treatment of choice. Proper counseling of the patient/family must be done, explaining them the prognostic factors and chances of disease recurrence.
